# A Qualitative Exploration of COVID-19 Vaccine Hesitancy Among Hispanic/Latine and African American and Black Middle-Aged and Older Adults in South Florida

**DOI:** 10.1089/heq.2022.0144

**Published:** 2023-03-10

**Authors:** Robbert J. Langwerden, Gladys E. Ibañez, María Eugenia Contreras-Pérez, Haby Abraham Contreras, Maria Luzardo Rivero, Sara C. Charles, Staci L. Morris, Adriana L. Campa, Eric F. Wagner, Michelle M. Hospital

**Affiliations:** ^1^ Community-Based Research Institute, Florida International University, Miami, Florida, USA.; ^2^ Research Center in a Minority Institution, Florida International University, Miami, Florida, USA.; ^3^ Department of Epidemiology, Florida International University, Miami, Florida, USA.; ^4^ Department of Dietetics and Nutrition, Florida International University, Miami, Florida, USA.; ^5^ Robert Stempel School of Social Work, Florida International University, Miami, Florida, USA.; ^6^ Institutional Review Board, Florida International University, Miami, Florida, USA.; ^7^ Department of Biostatistics, Florida International University, Miami, Florida, USA.

**Keywords:** COVID-19, disparities, focus groups, qualitative analysis, vaccine hesitancy

## Abstract

Racial and ethnic health disparities are more conspicuous in the United States since the start of the coronavirus disease 2019 (COVID-19) pandemic. While the urgency of these disparities was already alarming, the pandemic has exacerbated longstanding issues in health equity, disproportionate impacts, and social determinants of health. Vaccine hesitancy was a crucial factor during the U.S. COVID-19 vaccination campaign. We conducted a qualitative exploration of vaccine hesitancy through thematic analysis of four focus groups with Hispanic/Latine and African American/Black adults and senior citizens (*N*=23). The focus groups were conducted between February and April of 2021, in both English and Spanish. All participants (mean age=66.2, female 78.3%) were recruited by community-based organizations in the South Florida area. We explored six categories: (1) barriers to prevention and preventive behaviors, (2) barriers to vaccination against COVID-19, (3) facilitators of prevention and preventative behaviors, (4) facilitators of vaccination against COVID-19, (5) trusted sources of information, and (6) suggested macrolevel measures. These categories are discussed vis-à-vis COVID-19 disparities among racial and ethnic minorities. Implications for public health policy and future vaccination campaigns are outlined and discussed.

## Introduction

The coronavirus disease 2019 (COVID-19) pandemic continues to have a devastating impact on public health in the United States. While vaccines, booster vaccines, and massive public health campaigns to increase vaccine uptake have been instrumental, several variants and subvariants of COVID-19 (i.e., Delta, Omicron; BA.1, BA.2, BA.3, BA.4, BA.5) have emerged and caused the resurgence of cases, hospitalizations, and deaths.^1,2^ In the United States, Florida's population is, and at times, among the epicenters of U.S. COVID-19 infection.^3^ COVID-19 health disparities are a result of existing broader health disparities^4^ related to social determinants of health (SDOHs) such as social economic status (SES), socioeconomic factors, and stress due to discrimination and marginalization.^5^ SDOHs have been found to mediate health disparities in the United States overall^6^ and in South Florida,^7,8^ emphasizing the need for adequate assessment of personal factors and context when studying COVID-19 vaccine hesitancy among minority populations.^9^

Mid-to-late life adults is the age group among whom COVID-19 has caused the most hospitalizations and deaths,^10^ mental health problems,^11^ isolation, and negative effects on quality of life overall.^12^ This is particularly true for marginalized communities in the United States. Black or African American and Hispanic/Latine midlife and older individuals were found to have increased risks related to COVID-19, exposing existing health disparity mechanisms, such as structural racism (i.e., elevated odds of COVID-19 exposure, health care inequality, and weathering processes).^13^ National data indicate disparities exist also in COVID-19 trust and intention to get vaccinated,^14^ including among middle-aged and older adults,^15^ further worsening the risks among Black, Latine, and other minority middle-aged and older adults in the United States.^13^

In studying the context of COVID-19 impact and vaccination, as well as the unique stressors and socioeconomic burdens that minority populations face, qualitative investigations generally provide rich information and unique insight in the relationship between attitudes toward COVID-19, particularly on perceived COVID-19 vaccination barriers and facilitators,^16,17^ sources of information that are trusted to provide COVID-19 updates,^18^ and importantly, daily-life factors.^19^ Applying community-engaged principles and in partnership with community-based organizations, we conducted four focus groups with racial and ethnic minority middle-aged and elderly individuals to learn from their insights and perspectives on COVID-19 prevention, vaccination barriers and facilitators, and trusted sources of information. This knowledge can inform public health policy, information dissemination efforts, and removing barriers to COVID-19 vaccination.

## Materials and Methods

### Participants

Participants were recruited with the help of three community-based organizations that serve the communities in South Florida. Four focus groups were held virtually via a videoconferencing platform between February 1, 2021, and April 8, 2021. At the partnering CBO's request, two of the focus groups were conducted in Spanish and two were conducted in English. For an overview of the focus group participants' demographics, please see Table 1.

**Table 1.** Overview of Demographics for All Participants (*N*=23) and by Focus Group

Demographic	All	Group 1	Group 2	Group 3	Group 4
Age					
*M*	66.2	64.3	72.5	70	55.5
*SD*	9.9	6.8	12	3.6	13.9
Gender					
Female	18 (78.3%)	4	4	7	3
Male	5 (21.7%)	3	0	1	1
Race and ethnicity					
Hispanic/Latine and White	11 (47.8%)	7	0	0	4
Non-Hispanic/non-Latine and Black/African American^a^	12 (52.2%)	0	4	8	0

Group 1 and 4 were held in Spanish. Groups 2 and 3 were held in English.

^a^ One participant identified as Bahamian.

*M*, mean; *SD*, standard deviation.

### Materials and procedure

All participants provided verbal informed consent before participation in the focus group, as was approved by our institution's IRB on November 10, 2020 (approval number: IRB-20-0519). Study materials were developed by the research team and approved by our institution's IRB. Next, participants filled out a brief demographic form, which assessed age, gender, and racial/ethnic identification. They were given information on IRB confidentiality guidelines, such as instructions on keeping their video off, only using first names, and that the meetings would be audio recorded for research purposes. Groups lasted 60–90 min, facilitated by a trained moderator, and also had two research assistants taking notes. Participants received a $50 electronic gift card for their participation.

The focus group guides were originally drafted in English and included questions about (1) COVID-19 prevention and testing attitudes, knowledge, and beliefs, (2) trusted sources of information on COVID-19, (3) general pandemic attitudes, knowledge, and beliefs, (4) COVID-19 vaccine attitudes, knowledge, and beliefs, and (5) COVID-19 barriers, facilitators, and attitudes regarding COVID-19 accessibility and information dissemination. Study materials, including the guides and consent forms, were translated to Spanish. Independent research staff back translated the consent forms. The majority of research staff and authors are English/Spanish bilingual.

All focus group sessions were audio recorded and transcribed by three trained staff members, fluent in both English and Spanish. Staff members included one faculty member and two masters level graduate students. Spanish transcriptions were translated to English by fluent staff members. Once translated, the coding process consisted of a two-step approach. In the first step, three coders reviewed one of the focus groups and created a preliminary codebook of primary codes based on this group. All three coders reviewed and coded the rest of the focus groups using the preliminary codebook. Any new or emerging codes were subsequently added to the codebook. Any disagreements between coders were discussed until consensus was reached.

Using a deductive approach, the second step in coding focused on generating subcodes for each of the primary codes. For this step, coders worked in pairs. First, coders would code each primary code independently. Then, they would meet with the second coder to review and compare subcodes. Any disagreements were resolved through discussion. Each pair of coders further refined codes by discussing them with the third coder. Once subcodes were agreed upon, they were added to the codebook. After coding, data analysis utilized a thematic approach. That is, subcodes were analyzed for recurrent themes. Coders underwent substantial training on qualitative data analysis before coding and analysis. All coding and analyses were conducted using NVivo Pro Version 20.^20^

## Results

An overview of the recurrent themes within the research areas of interest—(1) barriers to protective behaviors, (2) barriers to vaccination, (3) facilitators of protective behaviors, and (4) facilitators of vaccination—are reported in Table 2. In addition, we also reported themes found with the categories of main sources of information and participant-suggested macrolevel measures to increase vaccination rates (not listed in Table 2). The themes displayed were generated from analysis of the focus group transcriptions. Each of the themes that emerged is discussed below.

**Table 2.** Overview of the Focus Group Themes Organized by Category and Subcategory

Barriers		Facilitators	
Protective behaviors	Vaccination	Protective behaviors	Vaccination
Main themes			
**PPE-related**	**Vaccine unavailability**	**General protective behavior**	**Vaccine services**
Having to go to the store	Age restrictions	Using masks, washing hands, etc.	Vaccination centers in neighborhood
Availability of PPE at stores	Location confusion	Social distancing	Organizations that help schedule appointments
PPE high prices	Appointment difficulties	Mask wearing/changing	Providing transportation
Use of masks improperly **Lack of information** Discrepancies with sources CDC information not clear Lack of guiding information Lack of technology access	Long lines Not enough centers **Structural/systems barriers** Lack transportation Immigration status Lack of doctor's approval No FDA approval **Fears/myths** Myths about vaccine Fear of side effects Fear of different vaccines Vaccine still being developed Concern about long term effects Vaccine rushed Fear of injected with COVID **Lack of information/misinformation** No guidance on how to take vaccine No information on vaccine No information on vaccine availability Vaccine discouraged on social media	Maintaining hygiene Double-masking or shield Wearing gloves Taking shoes/clothes off when home **Avoidance behavior** Avoiding crowds/large gatherings Avoiding busy places Avoid touching face Not allowing visits Staying home Avoid going to doctors Staying away from maskless people Staying within family only Staying in the car Not socializing **Cleaning behavior** Sanitizing kit in a car Use cleaning alcohol Sanitizing carts at stores	Setting appointments Open more centers Open vaccines to those without IDs Provide vaccines at large stores **Increase awareness** Show instructional videos More information on vaccine effectiveness Increase awareness about vaccine effects More information available **Social support for vaccination** Encouragement from family See leaders get vaccine See familiar people get vaccinated

CDC, Centers for Disease Control and Prevention; COVID, coronavirus disease; FDA, Food and Drug Administration; PPE, personal protective equipment.

### Barriers to protective behaviors

#### Personal protective equipment

Personal protective equipment will be referred to as PPE and was discussed under protective behaviors. Participants highlighted various barriers to protective behaviors against COVID-19 infection that were related to accessing PPE, including what PPE to purchase and how and where to purchase these. This provided helpful insight into what resources and information were available in the community. A participant was concerned with the in-person interactions, such as going to the grocery store to obtain PPE.

“But unfortunately, …. I have to go out to the store, and I don't like that, but I prefer the other way that I can go on and look at a list of things that I might need: alcohol, hydrogen peroxide, mask, gloves and it would come to my house.” (Female, African American, 69)

Another participant expressed dissatisfaction with the availability of PPE in the stores, as well as the high financial cost of PPE in general, including when purchased on the internet.

“…when I purchased them, I had to spend more money [than] I normally would have because I ordered them online for companies that were really had the prices up very, very high.” (Female, African American, 63)

Participants also discussed the misuse or lack of PPE use especially after vaccinations began as a barrier to being able to protect themselves.

“…people who don't properly wear their masks. When I do have to go out, I make sure I stay away from them, those that just want to put on a mask and have it underneath their nose an underneath the chin.” (Female, African American, 63)

#### Lack of information

Participants perceived that the Centers for Disease Control and Preventions (CDC's) guidance on what's regarded as protective behavior (and what was not) was not always clear. There were multiple participants who believed that there were discrepancies in the CDC's guidance.

“The CDC did not seem to step up in time to put the correct information and adequate information so everybody would be on the same page.” (Female, African American, 69)

In addition, there were references to a general lack of guiding information about what is protective.

“One of the things that we were able to do, and the lack of information that [the other participant] mentioned earlier it was a really, really sore spot for us because our seniors, and others … were not getting any information that [they] could use.” (Male, African American, 75).

### Barriers to vaccination

#### Vaccine unavailability

Apart from barriers to protective behaviors, such as access to PPE and information, there were specific questions about perceived barriers to COVID-19 vaccination. Participants discussed a wide range of barriers to getting vaccinated against COVID-19; either experienced by themselves or observed in others in their respective communities. One recurrent theme was the general unavailability of or difficulty in accessing the vaccine. Since several of the focus groups were conducted before all adults were eligible in Florida, age restrictions for the vaccine were referenced as a barrier at the time of the focus group.

Other perceived logistical barriers played a role in the participant's or participants communities' likelihood to get vaccinated, including confusion about locations of centers, not enough centers opened, and difficulties with appointments. The perceived lack of clarity regarding the location of the vaccination site as well as difficulty in making appointments for vaccination are evident in the subsequent quotes.

“So, they change their position, they say: from Monday to Tuesday here and when they go to another week, they already closed. And they tell you on TV, they're going to be on that location, so they've got people crazy.” (Female, Hispanic/Latine, 51)

“You can call all day and when they open up, like hospital opened up, they had, you know, they said their appointment were gone in less than an hour and it had over like 2000.” (Female, African American, 63)

#### Structural/systems barriers to vaccination

Several structural/systems related factors such as a lack of transportation and immigrant status were brought up as barriers to vaccination against COVID-19. Participants referenced SES and how that was linked to transportation as a challenge for getting vaccinated.

“Some of us, you know, have that capability, but a lot of times in the lower income community, transportation is a serious problem.” (Female, African American, 68)

Several participants referenced the perceived barrier of lack of documentation or their immigration status, such that people were not clear on what documentation was needed for vaccination. This included concerns about immigrants in their community who may lack documentation needed to make an appointment or receive the vaccine. Moreover, they referenced several mass distribution pharmacy chains that were perceived as requiring a valid ID or driver's license.

“I say it because I've heard it, on [pharmacy and supermarket websites], if you don't have a driver's license, they don't give it to you. Although the vaccine is there.…they say the vaccine is there, but we need your license. And then imagine, if that person doesn't have it, they're not going to get it … how do they get it?” (Female, Hispanic/Latine, 69)

One participant also noted confusion created by health care professionals. The doctor advised the participant against taking the vaccine due to the suspicion that the participant may have already been infected with the virus.

“I called my doctor and asked him, and he told me that as recently I had been with flu, last year, we still do not know. Also, we think that may[be] I had it. It seems, but he told me that for now, in my case, I shouldn't get it yet.” (Female, Hispanic/Latine, 66)

Finally, a general concern was the lack of access to technology, such that the information that was available was not necessarily accessible by many people in their community. The barrier this created was referenced to be a problem particularly for senior citizens.

“… that information is not as quickly accessible to all so not only was it not done timely with the appropriate coordination but the ability to share to large groups was limited because of the technology barriers that some of the seniors have.” (Female, African American, 66)

#### Fears and myths

Participants referenced a phenomenon in which people “waited to see what happens” during mass vaccination, such that people wanted to be reassured that the vaccine did not have any detrimental consequences. One participant expressed concern about potential detrimental consequences for the person's health, and worried that they would get infected with COVID-19 in the time they would be waiting.

“… there are a lot of people who don't want to take it because I hear people sitting there saying: you're crazy, leave, leave, let them give it to a lot of people to see what happens to them and then I'd get it.” I say yes, it is in the meantime when you're going to get [the virus].” (Female, Hispanic/Latine, 51)

More commonly, a general fear of side effects was prevalent among participants. One participant stated that this was heightened by the variability in side effects seen in different people. The idea that different people respond differently was referenced as fear-inducing.

“It may cause a reaction to me and not to the doctor. Do you understand? And that your arm does not hurt, and someone else's arm falls off. Some people get a headache, and others do not get a headache.” (Female, Hispanic/Latine, 66)

Amidst the spread of misinformation during the vaccine roll-out campaign, a common myth was referenced: the idea of being injected with a chip during vaccination.

“They say that it is a chip, that it won't be good for you.” (Female, Hispanic/Latine, 51)

Some participants noted that their fear was compounded by the confusion created by the availability of distinct types and manufacturers of vaccines, the urgency with which the vaccines were developed, as well as the different criteria (i.e., number of doses, timing of doses, eligibility criteria) that applied to several types.

“So, people are afraid of getting the vaccine, and they do not know what type of vaccine they are going to have because there are 3 or 4 different types of vaccines that are all for the same thing. However, there are many rumors among the population, and this is why most people are still very afraid to use the vaccine. Perhaps, if there were no discrepancies between the criteria that exist from one vaccine to another, the population would be more in agreement and more aware about acquire and administer this type of medicine.” (Female, Hispanic/Latine, 63)

Also, the same participant referenced a fear for the injection of live viruses for some types, which caused concern for people who were already taking medications, according to this participant.

“Viruses, most of the time, are live viruses introduced into the body, but other types of vaccines are not viruses. So, people are very afraid that once they use this medication, they may have an adverse reaction to what they are taking on.” (Female, Hispanic/Latine, 63)

#### Lack of information/misinformation

There was a general perceived lack of information regarding the vaccine, how to take the vaccine, where to go to take the vaccine, as well as misinformation on social media. One participant said that they needed guidance on how and when to take the vaccine, such that they could relay this information to others in their community.

“… I consider myself pretty astute about what's going on, but when they first started talking about the vaccine, I didn't have a clue to where even start.” (Female, African American, 68)

There was a reference to the lack of confidence among people participants knew, due to the lack of astute information available.

“I think lack of information—more information through the media, all kinds of media, with an explanation. I would get it, but I know people who say no because they are not confident because there has not been enough information for us to say: ok, I will get it and see the reactions.” (Female, Hispanic/Latine, 67)

### Facilitators of protective behaviors

#### General protective behavior

Participants mentioned general protective behaviors such as wearing masks, social distancing, wearing gloves, and taking clothes/shoes off when arriving home. This is shown in the following quotes.

“For me, what is being done, the distance and use of the mask, and washing your hands is easy.” (Female, Hispanic/Latine, 64)

“When I go out, if I go to the street or something, at first, I had shoes outside at the door, and I used to come into my house barefoot, took off my shoes, washed my hands well, washed my arms up to the top, and took off my clothes.” (Female, Hispanic/Latine, 66)

#### Avoidance behavior

Another recurrent theme referred to protective behaviors that include avoidant behavior such as avoiding crowds or gatherings, not allowing visits and avoiding family, staying away from maskless people, avoiding doctors, and staying home or in the car.

“Have the responsibility of knowing where you are and where you are going. You should not be in crowds because even if you have protection, the virus escapes because they are particles that infect.” (Male, Hispanic/Latine, 61)

“I hardly ever go anywhere or for instance, if I do go and get a car wash or something, I stay in the car. I don't let anybody get in or I'm just really trying to be really careful.” (Female, African American, 69)

#### Cleaning behavior

Other participants focused on protecting themselves through sanitizing or cleaning behavior.

“On the other hand, the most important thing for me is the mask, do not put your hands on your face and clean them with alcohol to avoid getting infected by hand contact. I think that's what has been working so far.” (Male, Hispanic/Latine, 61)

### Facilitators of vaccination

#### Vaccine services

Participants reported several suggested facilitators of vaccinations that referred to providing specific services. For example, participants mentioned opening more vaccine centers in the neighborhood, including at large stores known to the community.

“…like I said, there's a percentage, a large percentage where it would be more convenient for them to have it in their neighborhood.” (Female, African American, 69)

Other services include having organizations that help with scheduling appointments and transportation.

“Well, since some people do not have car to go, take twenty people on a bus with forty seats is an improvement. Or a vehicle like those of donations.” (Female, Hispanic/Latine, 66)

Finally, participants thought vaccines should be available to those without identification.

#### Increase awareness

Participants also thought that increasing awareness about the vaccine would facilitate vaccination in the community. Specifically, more information about the effectiveness of the vaccine was needed, and that showing instructional videos would be helpful.

“…I believe that to increase participation, the first thing is the information about raising awareness of the effects. If we don't use it, what are we doing? Instead of thinking for ourselves, it would be a way for people to be more optimistic about getting the vaccine.” (Male, Hispanic/Latine, 61)

#### Social support for vaccinations

Another recurrent theme was support for vaccinations among the participant's social circle or among respected leaders.

“…I said well, if they can do it and they're the ones running this country, now, why can't I do it?” (Female, African American, 70)

Finally, although not a recurrent theme, participants made references to community-based organizations, such as churches and civic organizations as facilitators to vaccination.

“[the church], they took their members down busloads at [local hospital] for them to have the shot.” (Female, African American, 90)

“I was able to assist my cousin in getting her appointment through another [Black] local civic organization.” (Female, African American, 63)

While not listed in Table 2, a secondary topic that continued to come up were main sources of trusted information and suggested macrolevel measures. We discuss both of these categories below.

### Main sources of information

These themes were related to perceived sources of information for the COVID-19 vaccine and about COVID-19 in general. Broad themes were mentioned, all of which are outlined below with illustrative quotes.

#### Community sources

First, churches were mentioned as important channels for information. Churches were also categorized under facilitators of information and seem to be an important theme in the perceived accessibility of COVID-19 vaccination information.

“…the churches have now become a vital asset because they are able to reach individuals from our medical and spiritual contacts which has encouraged a greater participation in the immunization drives for some of our residents.” (Female, African American, 66)

Another participant mentioned their workplace as an important source of information, since they get updated with COVID-19-related information multiple times a day.

“…I'm involved for a lot of things so therefore I get communications directly on daily basis, sometimes two or three times a day.” (Female, African American, 73)

Many physical communities, shops, such as supermarkets and pharmacies, were mentioned as disseminating resources.

“Also, in all shops and those parts, there is information on how to take care of ourselves.” (Female, Hispanic/Latine, 67)

On a larger scale, colleges and universities provided information on COVID-19 vaccines. In addition, their listservs and newsletter were said to be helpful and relied upon.

“Colleges have their, have their systems as well where they can media blast things.” (Female, African American, 66)

#### Family/friends as sources

Members of the family and friends were mentioned as important sources of information for the elderly. The value of conversations with family and friends was emphasized.

“Actually, with me, we as elders, you know who's like 65 and older, a lot of us talk to each other about the different things that have been going on with family members, with friends.” (Female, African American, 63)

#### Health care-related providers and organizations

Participants also mentioned larger, macrolevel sources of information that were useful. The CDC was mentioned as an outlet by a participant, particularly the CDC's website.^21^ Also, a card from the CDC was referenced, being mailed out in March of 2020.

“I get lots of information from my computer. I'm registered for emails from the CDC. When this virus first came out, they sent a card out from the CDC, in March of last year and they sent it to every American.” (Female, African American, 67)

Webinars with medical panels not only seemed to be important sources of information, but also a place where questions could be asked. Despite the challenges that technology brings for senior citizens, there seemed to be value in educational webinars and town halls (e.g., Wagner et al, 2022).^22^

“And they constantly have webinars, panels of health officials that can answer questions for you.” (Female, African American, 69)

Related to this, physicians and health organizations in general were mentioned by focus group participants. One participant mentioned having greater confidence in the information provided by medical experts.

“… the doctors. When I have the opportunity to talk with someone who has medical access, I always ask and receive information because I have greater confidence with those people.” (Male, Hispanic/Latine, 61)

#### Media sources

Apart from the outlets outlined above, including community-focused and large-scale efforts, various news outlets also were seen as helpful sources of information. The quotes below do not refer to any specific media source, but to the form or medium. Several participants mentioned radio stations to be useful, particularly local radio stations.

“The local radio stations they have: seminars and, you know, call in, and help people on that town to ask questions or acquire some of the misinformation you may have about the COVID.” (Female, African American, 69)

“The African American radio station in the Black community is phenomenal in terms of getting out information…is good and people like that when people have developed a relationship with radio; people are going out in the morning and just listen and he was going on in the community. But they are phenomenal.” (Female, African American, 63)

Television and news stations were discussed during the focus groups as beneficial outlets of COVID-19 information.

“I listen a lot to television. Through television I realize how things are, how everything is going, and I keep taking care of myself.” (Female, Hispanic/Latine, 64)

Websites also were referenced, and more specifically, search engines where one looks up symptoms or up to date information on COVID-19.

“I go on the Internet, basically. That's what I do and I look up COVID-19 symptoms. What is going on now. You know, I [online search] it and I just put in COVID-19 and what is going on with it.” (Female, African American, 68)

One participant mentioned social media as a tool to reach many people quickly. Facebook has been mentioned as a medium to reach many people quickly.

“Technology in terms of Facebook is a very good tool because you can reach many and not maybe few of them.” (Female, African American, 66)

Receiving information from someone of the same race or ethnicity and someone who is considered a leader in the community was mentioned as a beneficial avenue for information dissemination.

“[Information messages] need to be delivered by someone that the community has faith and confidence in.” (Female, African American, 73)

“He is on [radio station], he is, and in the African American community that the people have faith… he has been on the air providing informational news to people for years so they're custom to hearing him and they are custom to relying on him for solid information because sometimes if you bring up a politician that politician is about his or her.” (Female, African American, 73)

Finally, a participant mentioned that they got their information on COVID-19 and vaccination from the newspaper. In sum, there were references to a variety of local sources of COVID-19 information, such as churches and organizations and also local news outlets. The benefit of those outlets was emphasized in context of racial and ethnic minority elderly individuals and was trusted among the participants, such that these outlets could make an impact during a public health emergency.

### Macrolevel suggested measures

As a last category of themes, the focus group participants highlighted societal level or large-scale efforts that can be initiated to reach many people at once or to reduce vaccine hesitancy population-wise. Participants discussed a wide range of alternate measures or concrete steps that could be taken to support the population/community during the pandemic, protect the population/community from COVID-19, and reduce vaccine hesitancy and disseminate COVID-19 vaccine information.

#### Financial assistance

First, for general support, participants suggested that more financial support is needed.

“…If we're going to get rid of [COVID-19], they have to help financially. So that people don't have to go out.” (Female, African American, 63)

One participant suggested companies could incentivize people to get vaccinated by paying out added salary. This person acknowledged that businesses cannot mandate vaccinations in Florida.

“But what I was thinking too is: ask private companies to join, and those companies can incentivize people to get vaccinated. I don't know, tell him, well, look, we pay you half day, but we need you to get vaccinated. Because I know it's not mandatory, I know the vaccine can't be mandatory. But there's got to be some pressure where it's said: look, we need you to get vaccinated because we're going to infect people.” (Male, Hispanic/Latine, 38)

#### Protective measures

To protect people from being infected, participants mentioned several measures or strategies to avoid in-person contact. At several points, a preference for online shopping was discussed, or going to the store at nonbusy hours to avoid large numbers of people.

“I used to prefer the method of them sending everything that I needed to my house. And everything instead of going out.” (Female, African American, 68)

#### Dissemination of information

To reduce COVID-19 vaccination hesitancy (or increase confidence in the vaccines), several measures were suggested during the focus groups, including streamlined and efficient dissemination of information. One participant suggested tracking who is unvaccinated and another suggested disseminating information among them. A comparison was made to how information is disseminated for the census, as mentioned by a participant.

“… a database where they can use zip codes, send people to take data from people who have been vaccinated, check how many people live in zip codes.” (Male, Hispanic/Latine, 38)

“I think, look how they do for the census. Like, they mail you paperwork, they notify you wherever you want, you've already signed up.” (Female, Hispanic/Latine, 51)

Regarding information about the COVID-19 vaccines, there were comments about improving clarity on vaccine availability and eligibility, which addresses some of the confusion mentioned as barriers. This was emphasized by a participant who suggested local dissemination of vaccine information.

“… sometimes not relevant to what's going on here in Miami or what may be going on in Tallahassee or someplace in central Florida. I think there needs to be a local health group that will provide continuous reliable information on what is happening when it comes to COVID-19.” (Female, African American, 73)

By extension, the information was suggested to be in easy-to-understand vocabulary and refraining from using medical jargon.

“…more accessible words to each person because there are scientific words or other types of words, but many people do not have enough education to cover the terminology used to carry the message. I think it would be a more compact thing, but with words understandable by the population. That would be one of the most important things.” (Female, Hispanic/Latine, 63)

Given the multicultural and multilingual nature of South Florida's communities, a reference was made about the importance of translating information into different languages.

“And I would also think that if you can send us the information also in Spanish by mail.” (Female, Hispanic/Latine, 69)

Various concrete ways of information dissemination were suggested, such as giving people brief informational sessions at supermarkets and pharmacies in the community. This was in reference to the Hispanic/Latine communities.

“… use supermarket and popular or Latino pharmacies, where you can set people there, and explain to all people. Micro talks of 2–3 minutes, explain the benefits of the vaccine.” (Male, Hispanic/Latine, 38)

Furthermore, a mass-dissemination effort was suggested, in which text messages could be sent to many people. This leverages the fact that most people have a mobile phone as well as the importance of keeping the message brief.

“Get short messages to the phones, like when we get the Amber alerts when there is a lost kid or something. A few short messages.” (Female, Hispanic/Latine, 66)

A mass dissemination of COVID-19 vaccination flyers was also referenced, strategically placed at important places in the physical community, such as stores.

“What I was saying right now, put a sign on poles or in a specific place, on the stores' doors where people go…” (Female, Hispanic/Latine, 66)

Similar to canvassing for political campaigns, a participant suggested a door-to-door strategy. This participant stated that this could be more impactful than other efforts.

“This is an effective job because the flyers written in the street, on the poles, on the television in the newspaper or any of those things, it is not as effective as a door-to-door job.” (Female, Hispanic/Latine, 63)

Similarly, a participant suggested that supervisors notify their employees about vaccination opportunities, such as helping with vaccination appointments.

“Well, notifying people. Just as the supervisors have warned me about the vaccine, “Do you want to be vaccinated?”…So, I accepted, and they immediately told me, “I will call you with the appointment.” This is what they do with me.” (Female, Hispanic/Latine, 77)

Overall, the relevance of receiving this feedback from the community cannot be understated and can help inform future vaccination campaigns as well as other public health dissemination efforts.

## Discussion

We conducted a community-based qualitative exploratory study in partnership with organizations that serve middle-aged and older communities to investigate COVID-19 protection and vaccination facilitators and barriers. We also investigated trusted sources of information and solicited suggestions for macrolevel measures to protecting public health at large. We conducted focus groups with racial and ethnic minority individuals, of whom the majority was 65 years old or older. The focus groups were conducted to better understand perspectives on COVID-19 from these medically underserved and vulnerable communities on the COVID-19. This exploration is relevant for continued COVID-19 vaccination outreach campaigns as well as potential future public health campaigns with these communities.

Regarding barriers, we found that the participants mentioned both logistical and informational barriers. Logistical barriers, such as lack of technology and transportation issues, were often relevant to the middle-aged and older adult population. The participants were referring to and described a general fearfulness regarding in-person shopping or events. The findings also indicated that community members' perception of these logistical barriers was not only based on their own experiences but also others in their communities. These general logistical concerns are in line with findings from other studies, particularly one that found that these concerns are prevalent when vaccine availability is high.^23^

The barriers give insight into the unique struggles faced by the medically underserved in the South Floridian community, particularly the elderly community. Apart from logistical barriers, obstacles related to information access, information discrepancies, and misinformation were salient. An example of misinformation was a participant mentioning the injection of a chip when one gets vaccinated against COVID-19, a myth that was being spread when the vaccine was still in the development stage.^24^

Facilitators, motivators, and trusted sources of information in the context of COVID-19 vaccine and preventative measures were also explored in the focus groups. At multiple instances, participants referenced churches as being central to the community's information dissemination and vaccine campaigns. In the African American and Black communities in South Florida, churches have helped to get people vaccinated as well as by disseminating correct COVID-19 information and this was compared to how it might respond to a hurricane.^25^ The support of larger demographic-focused organizations, such as specific Black civic organizations and the AARP, were referenced as being relied upon for updated COVID-19 information. Websites, news, and email blasts from medical expert sources (including the CDC) also were named as being influential and accessed on a regular basis.

The role of family members, both in the context of helping with chores and other tasks in times of isolation as well as in the context of taking care of those by getting vaccinated, was understood to be important. For the participants, it was relevant to receive the information from someone they trusted, they mentioned family members, people from their church, or their community, and they included comments regarding receiving information from someone of the same race or ethnicity. These findings are congruent with other studies, and future research could build upon this by studying effective ways in which these channels could be leveraged to increase awareness and public knowledge.

The participants provided insight into what they thought would be effective or efficient ways to get others in their communities vaccinated. A wide array of strategies was discussed, including door-to-door canvassing, flyers, elevator pitches, and text messages. Many of the strategies discussed were later implemented by public health experts and authorities. The findings included suggestions to large efforts to reach the unvaccinated, such as sending mass text messages and reaching out by mail, like the census. In addition, the findings show the importance of physical locations in the community, such as supermarkets, stores, pharmacies, and churches. In an era in which digital marketing and information distribution are seen as crucial, the role of physical spaces in South Floridian Hispanic/Latine and African American or Black communities, particularly those that are frequented often, should not be overlooked. These findings may be taken into consideration for future vaccination campaign efforts.

Most importantly, the themes distilled from our transcripts are direct representations of what community members find best for their communities and inform how future efforts can be tailored to these communities. On another level, front line providers and policy makers may be better equipped to help these communities when learning about these contexts, motivators, and most importantly, barriers. For example, the importance of churches as an outlet of information emerged as a recurring theme, which can be useful information for on-the-ground vaccination teams and staff. Next, municipalities may learn from these results the wide range of experiences during the pandemic retrospectively. It is important to note that not all communities within a municipality, particularly those as large as Miami-Dade, are homogenous and that barriers and facilitators may be community-specific. On the local, state, and national level, policy makers should be sensitive to these community level distinctions.

Finally, investigators may be reminded that listening to community members' qualitative feedback can provide crucial information and contextualization. The results of this study highlight that although surveys and quantitative assessments have their benefits, more qualitative research is needed that can provide greater insight into unique experiences of community individuals.

The current study had several limitations. First, the reliance upon video conferencing as the medium for the focus groups may have limited or thwarted the participation of some older adult community members. The reliance upon technology during the COVID-19 pandemic was specifically referenced to be a barrier during the focus groups. Second, we also must consider that the circumstances of the COVID-19 pandemic are changing by the day. Therefore, these data need to be interpreted in light of its pertinence in the relative context. When the focus groups were conducted, vaccines were only available for adults aged 65 years and older, and some of the macrolevel suggested measures have already been implemented.

Even so, we believe that it is pertinent to present these data since they are part of a story that contains insights by medically underserved and vulnerable typically underrepresented minorities. These insights are often underemphasized and individuals from underserved backgrounds rarely get to share perspectives. The specific experiences of the Hispanic/Latine and Black or African American adults in South Florida can provide unique insight that may allow us to better prepare informative campaigns to raise awareness regarding COVID-19 or other deceases.

Several strengths warrant mentioning as well, including the community-based nature of this study. We owe thanks to the community partners who helped recruit participants from diverse communities in the South Florida metropolitan area. We were able to recruit mid-to-older-aged adults from racial and ethnic backgrounds amidst a complicated and fearful time. Our partners facilitated the access to technology that otherwise would not have been available to this elderly groups. While the reliance upon technology is a limitation, it helped anonymize data collection and protect participants' confidentiality while also protected them from COVID-19 by not having to meet in person. Moreover, we wanted to center the community members' voices in this research study, in line with the aims of the Florida Community Engaged Alliance (FL-CEAL) Against COVID-19 in Disproportionately Affected Communities.^26^

We did this by 1) recruitment through trusted community organizations in South Florida, 2) designing a focus group manual that prioritizes participants' opinions using open, nonsuggestive language, and 3) conducting Spanish focus groups for participants who preferred this. The insights provided in this article can be used to inform and shape policy around vaccination efforts regarding COVID-19, other diseases, and other public health emergencies.

In sum, our community-engaged qualitative exploration on COVID-19 barriers, facilitators, trusted sources of information, and suggested macrolevel measures evidenced a multitude of themes at the start of the COVID-19 vaccination campaign in South Florida. We want to emphasize the unique perspectives (summarized in Table 2) that community members provided regarding their respective communities, and yet, we do not claim these to generalize to other geographical areas.

## Abbreviations Used

CDC: Centers for Disease Control and Prevention
COVID-19: coronavirus disease 2019
FDA: Food and Drug Administration
*M*: mean
PPE: personal protection equipment
*SD*: standard deviation
SDOHs: social determinants of health
SES: social economic status
